# Generalised Periodontitis: Examining TAS2R16 Serum Levels and Common Gene Polymorphisms (rs860170, rs978739, rs1357949)

**DOI:** 10.3390/biomedicines12020319

**Published:** 2024-01-30

**Authors:** Albertas Kriauciunas, Greta Gedvilaite, Akvile Bruzaite, Gediminas Zekonis, Dainius Razukevicius, Rasa Liutkeviciene

**Affiliations:** 1 Department of Prosthodontics, Lithuanian University of Health Sciences, Sukilėlių Str. 51, LT-50106 Kaunas, Lithuania; gediminas.zekonis@lsmu.lt; 2 Laboratory of Ophthalmology, Institute of Neuroscience, Medical Academy, Lithuanian University of Health Sciences, Eivenių Str. 2, LT-50009 Kaunas, Lithuania; greta.gedvilaite@lsmuni.lt (G.G.); akvile.bruzaite@lsmu.lt (A.B.); rasa.liutkeviciene@lsmu.lt (R.L.); 3 Department of Oral and Maxillofacial Surgery, Lithuanian University of Health Sciences, Eivenių Str. 2, LT-50161 Kaunas, Lithuania; dainius.razukevicius@lsmu.lt

**Keywords:** TAS2R16, polymorphisms, serum levels, periodontitis

## Abstract

The objective of this study was to evaluate and compare the associations between TAS2R16 serum levels and common gene rs860170, rs978739, and rs1357949 polymorphisms in patients affected by generalized periodontitis. The study enrolled 590 patients: 280 patients with periodontitis and 310 healthy controls as a reference group. Patients underwent periodontal examination and radiographic analysis to confirm the periodontitis diagnosis. Blood samples were collected, and the DNA salting-out method was used for DNA extraction from peripheral venous blood. Genotyping of *TAS2R16* (rs860170, rs978739, and rs1357949) was performed using real-time polymerase chain reaction (RT-PCR), and serum level analysis was performed for both periodontitis-affected patients and reference group subjects. The analysis of *TAS2R16* rs860170 (TT, CT, and CC) showed a statistically significant difference between generalized periodontitis and the reference group (41.8%, 58.2%, and 0% vs. 38.7%, 56.1%, and 5.2%, *p* < 0.001). *TAS2R16* rs860170 (TT, CT, and CC) showed a statistically significant difference between males in generalized periodontitis and reference groups (38.4%, 61.6%, and 0% vs. 32.9%, 56.6%, and 10.5%, *p* = 0.002). Female-specific analysis showed that the *TAS2R16* rs978739 C allele was more frequent in generalized periodontitis compared to the reference group (37.5% vs. 28.7%, *p* = 0.016). Subjects aged 70 years and older demonstrated a statistically significant difference in *TAS2R16* rs860170 (TT, CT, and CC) between generalized periodontitis and the reference group (42.8%, 57.2%, and 0% vs. 38.6%, 53.8%, and 7.6%, *p* = 0.003). TAS2R16 serum levels were elevated in generalized periodontitis compared to the reference group (0.112 (0.06) ng/mL vs. 0.075 (0.03) ng/mL, *p* = 0.002). Females carrying the *TAS2R16* rs978739 C allele were more prone to generalized periodontitis development. Associations were found between *TAS2R16* rs860170 polymorphisms, elevated TAS2R16 serum levels, and generalized periodontitis development.

## 1. Introduction

A significant global population of 3.60 billion individuals experiences the impact of chronic oral conditions that involve untreated dental caries, periodontal disease, and complete tooth loss [1]. Specifically, severe periodontitis affects approximately 11.2% of the global population, ranking it as the sixth most prevalent human disease and is classified as a chronic non-communicable disease (NCD) that exhibits a considerable prevalence [2]. This condition is a chronic inflammatory disease that arises from a combination of multiple factors. It is closely linked to the presence of an imbalanced biofilm and is characterized by the gradual deterioration of the structures that support the teeth. In severe cases, this deterioration can ultimately result in the loss of affected teeth and not only affects oral health but is also linked to other systemic conditions, including diabetes mellitus, cardiovascular disorders, and rheumatoid arthritis [3,4]. According to the American Dental Association (ADA), oral health encompasses various dimensions, including functionality, structure, aesthetics, physiology, and psychology. It is seen as a fundamental aspect of an individual’s overall health and contributes significantly to their quality of life [5].

Historically, *TAS2R16* genes have been subject to balancing selection for an extended period prior to the appearance of modern humans in Africa. Their primary function was likely to prevent the consumption of hazardous, uncooked foods, focusing on a broad range of bitter sensations [6].

The TAS2R16 gene encodes a member of the G protein-coupled receptor superfamily, which is a family of potential taste receptors. These family members are uniquely detected by taste receptor cells located in the tongue and palate epithelia. Each of these genes within introns encodes a seven-transmembrane receptor protein, serving as a bitter taste receptor [7]. The prototypical bitter taste receptor TAS2R16 is known to respond to ~30 different β-glucoside compounds [8]. This gene is co-located with three other potential taste receptor genes on chromosome 7 and is genetically associated with regions that affect the experience of bitterness [7].

The association between periodontitis and gene polymorphisms was a point of research for a long time in both genetics and dentistry. Modern investigation of TAS2R expression in various tissues and cell lines has been conducted through the utilisation of RT-PCR, qPCR, microarray techniques, and RNAseq [9]. The initial identification of T2Rs occurred within the oral cavity’s taste buds [10]. TAS2R (taste receptor type 2) family—which are G-protein-coupled receptors involved in bitterness sensing mechanisms [7,11]. They act as sentinels in protecting against the ingestion of potentially toxic substances [12]. The TAS2R gene family consists of 11 pseudogenes and 25 full-length genes [13]. The activation of TAS2Rs has been observed to counteract the production of inflammatory mediators caused by lipopolysaccharide (LPS) in both human whole blood and lung macrophages. This suggests that TAS2Rs may play a significant role in effectively regulating inflammation [14]. In the TAS2R family, the TAS2R16 gene is responsible for the receptor-mediating response to beta-glucopyranosides [15,16]. It is also known that TAS2R genes are expressed differently, and TAS2R16 is one of the barely detectable genes [17]. Numerous investigations, covering cell culture experiments and human and animal studies, have been conducted to explore the involvement of taste receptors in innate immunity [18,19,20,21]. According to scientific findings, bitter taste receptors have been found to be expressed in cell types that do not primarily participate in oral sensory perception [22]. Although limited studies were conducted associating *TAS2R16* with periodontitis, some links were found by investigating various gene–environment interactions in the development of dental caries and observing that specific variations in *TAS2R38* genes were linked to an increased susceptibility to dental caries when exposed to particular environmental factors [23]. Some studies regarding the same gene have indicated that the *TAS2R38* genotype has a significant role in modulating the reaction of gingival epithelial cells to bacteria associated with both caries and periodontal disease [24]. Other investigations have identified T2Rs as possible targets for therapeutic intervention in order to modify innate immune responses during oral bacterial infections [25]. Nucleotide polymorphisms located in the area of the coding exon of TAS2R16 have been identified as potential risk factors for the development of alcohol and nicotine dependence among individuals of African-American descent [26]. Nevertheless, there is a lack of scientific literature regarding the mechanisms and biological importance of the anti-inflammatory effects caused by TAS2Rs in cases of periodontitis.

The aim of this research was to analyse the TAS2R16 serum levels and common gene rs860170, rs978739, and rs1357949 polymorphisms in patients affected by generalized periodontitis.

## 2. Materials and Methods

### 2.1. Ethical Approval and Data Protection

The study was conducted at the Laboratory of Ophthalmology, Neuroscience Institute, Medical Academy, Lithuanian University of Health Sciences (LUHS), Kaunas, Lithuania, and the Department of Prosthodontics, LUHS, Kaunas, Lithuania. The study protocol was confirmed by the Kaunas Regional Ethics Committee for Biomedical Research (authorization number BE-2-20). The participants were introduced to the structure and objectives of the study before its execution. An informed consent form was obtained from all subjects involved in the study.

### 2.2. Materials

#### 2.2.1. Study Group

The study enrolled 590 subjects: 280 PD patients and 310 reference group subjects. The characteristics of the study subjects are described in Table 1. The clinical characteristics of periodontitis patients and healthy subjects are described in Table 2. The data of age and gender were compared between the PD and control groups. The reference group was adjusted by age and gender to the PD group (*p* = 0.105; *p* = 0.133, respectively).

#### 2.2.2. Inclusion and Exclusion Criteria

The inclusion criteria for periodontitis-affected patients for this study were in accordance with the “2017 Classification of Periodontal and Peri-implant Diseases and Conditions” classification of periodontal diseases [27]:

- Patients age > 18 years old.
- Patient’s informed and voluntary consent to participate in the study, which included radiographic and intraoral periodontal examination to determine the extent of periodontal disease.
- Generalized periodontitis of stages III and IV—more than 30% of the patient’s oral region affected by periodontitis (examined on their first visit to either the prosthodontist or periodontist.
- Radiographic evidence of bone loss.
- Interdental clinical attachment loss ≥ 5 mm (III–IVth stage periodontitis).
- Tooth loss due to periodontitis.
- Vertical bone loss ≥ 3 mm.
- <20 remaining teeth (10 opposing pairs).
- Probing depths ≥ 6 mm.

Inclusion criteria for the reference group individuals for this study were:

- No bleeding on probing (BOP).
- No clinical signs of gingiva inflammation.
- No clinical attachment loss was present, and probing depth was ≤3 mm.
- No previous history of periodontal diseases.

The exclusion criteria for both groups (periodontitis and reference) of this study were:

- Patients who were undergoing orthodontic treatment.
- Diabetes mellitus-affected patients.
- Patients with any medical records of chronic inflammatory diseases, HIV, hepatitis, autoimmune disorders.
- Pregnant or breastfeeding patients.
- Patients who had any type of infection that required antibiotic treatment in the last 3 months.
- Patients under chemotherapy treatment (active or history of chemotherapy).

Intraoral periodontal and radiographic examinations took place during the patient’s first visit to the prosthodontist or periodontist. The diagnosis of periodontal disease was determined according to the consensus report of Working Group 2 of the World Workshop on the Classification of Periodontal and Peri-Implant Diseases and Conditions 2017 [27].

#### 2.2.3. DNA Extraction

DNA extraction was performed at the Laboratory of Ophthalmology, Neuroscience Institute, Medical Academy, LUHS. The DNA was extracted from peripheral venous blood samples using the DNA-salting-out method. To isolate DNA from blood samples, 3 mL of blood was transferred into 15 mL centrifuge tubes. Subsequently, 6 mL of cold lysis buffer, a crucial component in DNA extraction responsible for breaking down cell membranes and nuclear envelopes and facilitating the release of cellular contents, including DNA, was added. The mixture was then centrifuged at 3000× *g* for 6 min. After centrifugation, the supernatant was carefully removed, leaving only the sediments. This process was repeated 4–5 times to eliminate any remaining red blood cells. Following the final centrifugation, the supernatant was discarded, and the tubes were briefly inverted on a paper towel. To the sediments, 2.4 mL of cold lysis II, 150 µL of 10% SDS, and 5 µL of proteinase K were added, followed by gentle mixing. The mixture was then incubated at 56 °C for 10 min. After incubation, 1 mL of NaCl (6 M) and 1 mL of cold chloroform were added, and the solution was thoroughly shaken before centrifugation at 3000× *g* for 5 min. Upon centrifugation, a bi-phase fluid was observed, and the supernatant was carefully transferred to new centrifuge tubes. To the new tubes, 96% ethanol (1:1 ratio) was added, precipitating the DNA. The precipitated DNA was transferred to Eppendorf tubes with 70% ethanol, followed by centrifugation at 17,000× *g* for 1–2 min. After removing the ethanol, the DNA was retained, and the tubes were placed in an incubator at 37 °C until the alcohol evaporated. Finally, 100 µL of TE buffer was added, and the tubes were left at room temperature or in the fridge for 30 min. The spectrophotometric evaluation of DNA concentration and purity was conducted using a spectrophotometer (Agilent Technologies, Cary 60 UV–Vis, Santa Clara, CA, USA).

### 2.3. Methods

#### 2.3.1. Genotyping

*TAS2R16* gene rs860170, rs978739, and rs1357949 polymorphisms were genotyped using the real-time polymerase chain reaction (PCR) method. All single-nucleotide polymorphisms (SNPs) were identified using TaqMan^®^ Genotyping assays (Thermo Fisher Scientific, Inc., Pleasanton, CA, USA). The genotyping was performed using a “StepOnePlus” real-time PCR quantification system (Thermo Fisher Scientific, Singapore). The results of individual genotypes were obtained using the Allelic Discrimination program during the real-time PCR. A retesting was conducted on a random sample, constituting 5% of the entire DNA sample, to validate the results and ensure the consistency of the genotyping procedure. The genotype call rate was determined to be 99.575%, indicating a high proportion of successfully determined genotypes. Furthermore, the agreement rate, representing the percentage of agreement between duplicate samples or repeated assays, was exceptionally high at 99.899%. These values underscore the precision and reliability of our genotyping procedures in this study.

#### 2.3.2. TAS2R16 Serum Levels Measurement

*TAS2R16* serum levels were evaluated in 20 reference group subjects and 20 PD patients. The assay was performed using the Abbexa ELISA Kit for Human Taste receptor type 2 member 16 (TAS2R16), standard curve sensibility range: 0.312 ng/mL–20 ng/mL, sensitivity < 0.1 ng/mL, following the manufacturer’s instructions, and analysed on the Multiskan FC Microplate Photometer (Thermo Scientific, Waltham, MA, USA) at 450 nm.

#### 2.3.3. Statistical Analysis

The data about the study participants’ demographic characteristics were compared between the reference group subjects and the PD group using the Pearson chi-square test and Mann–Whitney U test and presented as absolute numbers with percentages in brackets. The frequencies of all selected *TAS2R16* SNP genotypes and alleles are shown in absolute numbers with percentages in brackets.

Chi-square test was used to compare the distribution of *TAS2R16* SNPs in the PD and reference groups. Binary logistic regression analysis with an adjusted odds ratio (OR) and its 95% confidence interval (95% CI) was performed to evaluate the influence of the *TAS2R16* association with PD occurrence. The binary logistic regression analysis results are represented as genetic models: Codominant, dominant, recessive, overdominant, and additive. The best genetic model selection was based on the Akaike Information Criterion (AIC), where the best genetic model was the one with the lowest AIC value. IBM SPSS Statistics (Statistical Package for the Social Sciences) Version 29.0 software was used for comprehensive data analysis. In the analysis, the “SNPStats” program was employed to examine haplotypes. The study also involved assessing linkage disequilibrium among the gene polymorphisms under investigation. Calculations included determining the deviation between expected and observed haplotype frequencies (D′) and evaluating the square of the correlation coefficient of haplotype frequencies (r2).

After Bonferroni correction, the findings were considered statistically significant at *p* < 0.05/3 (*p* < 0.017). Only statistically significant variables are in bold.

## 3. Results

In the present study, we genotyped *TAS2R16* rs860170, rs978739, and rs1357949 SNPs and analysed possible associations between selected SNPs and PD development. Table 3 lists the frequencies of selected SNP genotypes between the PD group patients and reference group subjects. *TAS2R16* rs860170 (TT, CT, and CC) showed a statistically significant difference between PD and the reference group (41.8%, 58.2%, and 0% vs. 38.7%, 56.1%, and 5.2%, *p* < 0.001) (Table 3). However, binary logistic regression analysis did not reveal statistically significant results (Table 4).

Also, we calculated the Hardy–Weinberg equilibrium (HWE) *p*-value by comparing observed genotype frequencies in our study population with expected frequencies, assuming genetic equilibrium. HWE test results indicated that genotypes of TAS2R16 rs978739 and rs135794 in the reference group did not deviate from HWE (*p* >0.05). However, *TAS2R16* rs860170 deviated from HWE (Table 3). Despite this deviation, we chose not to exclude this polymorphism from further statistical analysis, as the observed genotype and allele distribution may be attributed to the small sample size. We acknowledge the need to increase the sample size in future studies.

Gender-specific analysis was conducted, and when examining the male group, genotype and allele distribution analysis revealed the same results as before: *TAS2R16* rs860170 (TT, CT, and CC) showed a statistically significant difference between males in PD and reference groups (38.4%, 61.6%, and 0% vs. 32.9%, 56.6%, and 10.5%, *p* = 0.002) (Table 5). However, no statistically significant results were found after binary logistic regression analysis in the male group (Table 6).

Female-specific analysis showed that the *TAS2R16* rs978739 C allele was more frequent in PD compared to the reference group (37.5% vs. 28.7%, *p* = 0.016) (Table 7).

To evaluate the impact of *TAS2R16* rs860170, rs978739, and rs1357949 on PD occurrence in the female group, a binary logistic regression was applied. However, the results did not show a statistically significant impact of selected SNPs on PD occurrence after the Bonferroni correction (Table 8).

Age-specific analysis showed no statistically significant findings when examining subjects under the age of 70 (Table 9 and Table 10).

Age-specific analysis, when subjects were aged 70 years and older, demonstrated a statistically significant difference in *TAS2R16* rs860170 (TT, CT, and CC) between PD and the reference group (42.8%, 57.2%, and 0% vs. 38.6%, 53.8%, and 7.6%, *p* = 0.003) (Table 11). However, binary logistic regression analysis did not reveal statistically significant results (Table 12).

**Figure 1:**
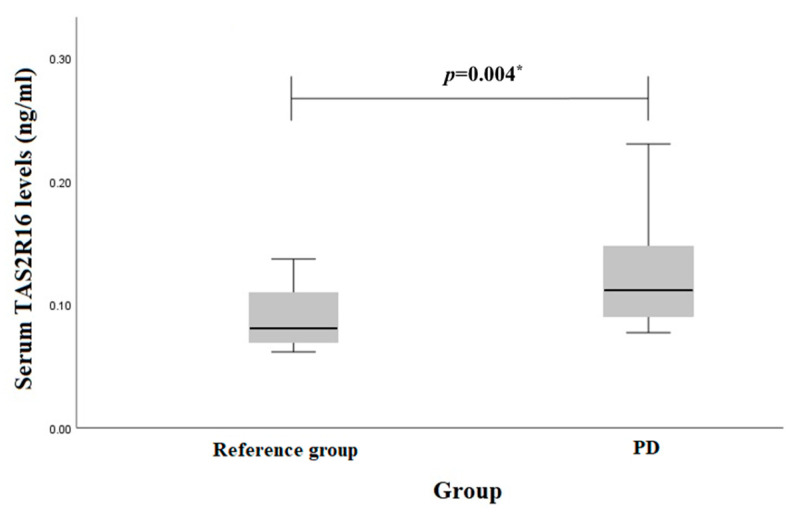
Serum *TAS2R16* levels (ng/mL) in PD and reference groups. * Mann–Whitney U test was used.

TAS2R16 serum levels in PD patients and reference group subjects were evaluated. We found that TAS2R16 serum levels were elevated in PD compared to the reference group (0.112 (0.06) ng/mL vs. 0.080 (0.04) ng/mL, *p* = 0.004) (Figure 1).

**Figure 2:**
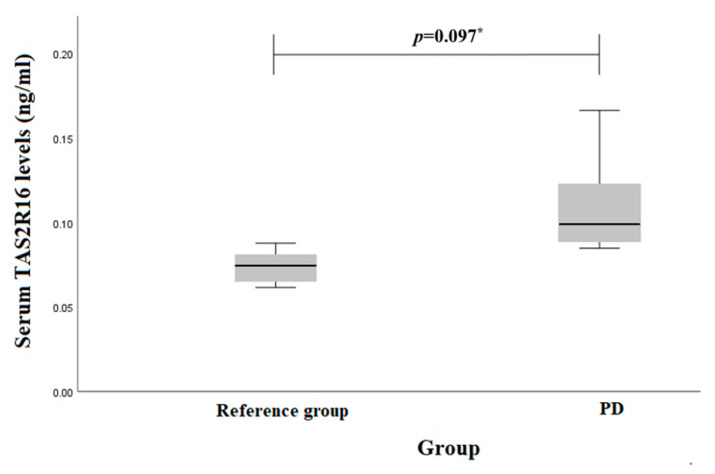
Serum *TAS2R16* levels (ng/mL) in PD and reference group females. * Student *t* test was used.

**Figure 3:**
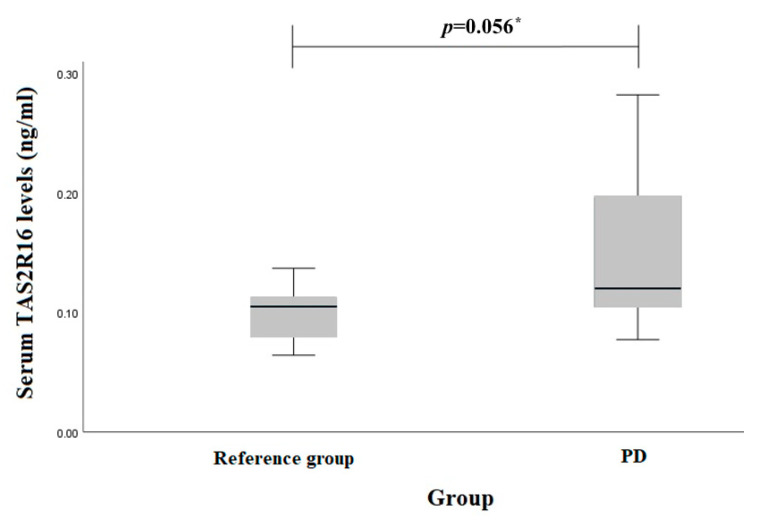
Serum *TAS2R16* levels (ng/mL) in PD and reference groups males. * Student *t* test was used.

TAS2R16 serum levels in PD patients and reference group females and males were evaluated separately. However, there were no statistically significant results between females (0.110 (0.03) ng/mL vs. 0.084 (0.04) ng/mL, *p* = 0.097) (Figure 2) or males (0.149 (0.07) ng/mL vs. 0.101 (0.02) ng/mL, *p* = 0.056) (Figure 3).

A comparison of serum TAS2R16 levels between different genotypes for selected single nucleotide polymorphisms was performed. PD patients with the CT genotype of *TAS2R16* rs860170 exhibited higher serum TAS2R16 levels than reference group subjects (*p* = 0.031). Similarly, PD patients with the CT genotype of *TAS2R16* rs978739 had elevated serum TAS2R16 levels compared to reference group subjects (*p* = 0.022). However, these results did not reach statistical significance after Bonferroni correction (Table 13).

Also, haplotype association analysis of *TAS2R16* rs860170, rs978739, and rs1357949 was performed in PD patients compared with a reference group. The pairwise linkage disequilibrium between SNPs is shown in Table 14.

Statistical analysis showed that individuals carrying haplotypes T-T-A, C-T-A, and C-C-A of SNPs rs860170, rs978739, and rs1357949 were associated with 1.9-fold, 5.6-fold, and 6.3-fold decreased odds of PD occurrence (OR = 0.53; 95% CI: 0.34–0.81, *p* = 0.003; OR = 0.018; 95% CI: 0.07–0.49, *p* < 0.001; OR = 0.16; 95% CI: 0.05–0.56, *p* = 0.004, respectively) (Table 15).

## 4. Discussion

In this study, we aimed to analyse the association between *TAS2R16* rs860170, rs978739, and rs1357949 polymorphisms and *TAS2R16* serum levels and patients affected by generalized periodontitis, in comparison to a control group of healthy subjects.

Although no scientific literature describing *TAS2R16* (rs860170, rs978739, and rs1357949) polymorphisms in association with periodontal diseases was found, numerous extra-gustatory tissues, including the respiratory tract, gastrointestinal mucosa, and gingiva, have been shown to express TAS2Rs [28,29,30]. Besides being involved in taste perception, TAS2R-expressing extra-gustatory cells also serve as immune system sentinels in the innate immune response of mammals. TAS2Rs have a wide range of sensitivity to bacterial chemicals and are involved in several functions, such as nitric oxide synthesis, cilia beating, the establishment of type 2 immunity, and direct bactericidal effects in the airways and gut [31].

Furthermore, research has recorded the involvement of TAS2R activation in exerting anti-inflammatory effects through the inhibition of cytokine expression in various cells or tissues [32]. Zhou et al.’s study demonstrated that salicin effectively inhibited the expression of proinflammatory cytokines, such as IL-6 and IL-8, induced by LPS. This effect was achieved through the activation of TAS2R16. Neutrophils were found to be the primary type of leukocytes recruited to the periodontium. Additionally, the study identified and analysed the expression of TAS2Rs in HGFs and discovered the involvement of calcium signalling in response to different bitter flavours. Furthermore, salicin, a particular activator of TAS2R16, demonstrated an anti-inflammatory effect by suppressing the cAMP and NF-κB signalling pathways in a TAS2R16-dependent manner. Hence, our data indicate the potential use of TAS2Rs as a pharmacological target for addressing periodontitis [14]. Also, a prior investigation demonstrated that administering the bitter chemical denatonium benzoate to mice with periodontitis resulted in an increased production of antimicrobial peptides by gSCCs and hindered bacterial colonisation. As a result, this treatment effectively reduced alveolar bone loss associated with periodontitis [33]. Hence, clinical research has demonstrated that gene variants of TAS2R38 are associated with a defensive impact against dental caries [34]. TAS2R38 is a crucial factor in protecting the sinonasal epithelium and has a role in bacterial infections of the respiratory system [35]. Considering the extensive distribution of TAS2Rs and their physiological roles, it is feasible to utilise bitter compounds for therapeutic purposes. TAS2Rs may have a pivotal role in the pharmacological effects of herbal medications [36]. Berberine, which acts as an agonist for TAS2R38 and TAS2R46, has the ability to decrease inflammatory reactions and has been used for a long time in treating inflammatory bowel disease [37]. Scutellaria baicalensis Georgi, a frequently utilised herbal remedy, exhibits anti-inflammatory properties when employed for the treatment of respiratory and intestinal ailments [38]. The bioactive components of Scutellaria baicalensis Georgi, namely baicalin, baicalein, and wogonin, have the ability to activate TAS2R14 as bitter agonists [38,39].

Due to the significant role of a strong immune response in promoting a long lifespan, it is expected that a specific variation in the genes responsible for bitter taste receptors, specifically TAS2R16, TAS2R4, and TAS2R5, is linked to longevity [40]. This association was observed in a population of 941 individuals ranging from 20 to 106 years old in Calabria, Italy. Following adjustment for multiple testing, only one single nucleotide polymorphism (SNP), rs978739, had a statistically significant correlation with lifespan (*p* = 0.001). Specifically, the proportion of individuals with the homozygous A/A genotype grew gradually from 35% in individuals aged 20 to 70 years to 55% in individuals who reached the age of 100 [41]. Carrai and colleagues conducted an unconditional logistic regression analysis, taking into account age, gender, and nationality. They found that persons who had at least one A allele of the TAS2R16—rs6466849 gene had a reduced tendency to drink wine, although this reduction was not statistically significant. The odds ratio was 0.65 (95% confidence interval: 0.40–1.04, *p* = 0.071). When we divided the subjects based on gender, we found that the connection between wine drinking and the outcome was statistically significant only in females (*p* = 0.005). This suggests that there may be an interaction between gender and wine consumption [42].

Based on this knowledge, it could be hypothesised that TAS2R16 could modulate chronic inflammation in the gut [43]. To perform our analysis, we selected three SNPs (rs860170, rs978739, and rs1357949) that mark the most frequent variants in the analysed gene (*TAS2R16*) region. The selection was mainly based on the linkage disequilibrium results between all variants in the region. In Zhou and colleagues’ study, 22 subtypes of taste receptor family 2 (TAS2Rs) and the downstream mechanisms of Gα-gustducin and phospholipase C-β2 (PLCβ2) were identified in human gingival fibroblasts (HGFs). Various bitter agonists were able to induce an intense cytosolic Ca^2+^ response in HGFs. More importantly, TAS2R16 was expressed at a relatively high level, and its agonist, salicin, showed robust Ca^2+^-evoking effects in human gingival fibroblasts (HGFs). Activation of TAS2R16 signaling by salicin inhibited the release of lipopolysaccharide (LPS)-induced proinflammatory cytokines, at least in part, by suppressing LPS-induced intracellular cAMP elevation and NF-κB p65 nuclear translocation in HGFs [14]. These results suggest that activation of TAS2Rs in HGFs may mediate the elimination of endogenous pro-inflammatory processes by antagonising NF-κB signalling, providing a new paradigm and treatment target for the improved treatment of periodontitis.

Various studies were analysed for predicting the role of TAS2R16 rs860170, rs978739, and rs1357949 polymorphisms in biological pathways associating with various other pathological conditions. A study conducted by Risso et al. revealed that the perception of salicin bitterness was linked to the presence of the TAS2R16 rs860170 A allele (adjusted *p* = 0.01) [44]. Barontini et al. conducted a study that concluded that the TAS2R16 rs860170 polymorphism did not have a significant impact on the susceptibility to colon cancer [43]. A study by Inokaityte et al. revealed that the presence of the C allele of TAS2R16 rs860170 was linked to a 2.8-fold higher likelihood of developing exudative AMD in women and a 2.9-fold higher likelihood in men [45]. On the other hand, Malovini et al. did not find any indication of a connection between TAS2R16 rs978739 and the longevity phenotype, regardless of whether the additive or dominant model was considered [46]. Also, the study by Campa et al. has shown that the haplotype (rs1357949–rs6466849–rs860170–rs978739: T_A_A_G) of the TAS2R16 gene exhibited a suggestive correlation with longevity [41]. Another study by Clark et al. investigated the TAS2R42 SNP rs1357949 association with dysregulation of thyroid hormones triiodothyronine and thyroxine (T3/T4), although the results showed no statistically significant association for the aforementioned polymorphism [47]. A study conducted by Dotson et al. examined the impact of TAS1R- and TAS2R-type taste receptors on glucose homeostasis. The findings revealed several genetic variations linked to changes in glucose and insulin regulation. However, the rs1357949 polymorphism did not demonstrate any statistically significant association with the study results [48].

Some studies have reported a link after a cell culture investigation was conducted to examine the relationship between taste genotypes *TAS2R43* and *TAS2R50*, as well as artificial bitter compounds. The findings of this study revealed that the *TAS2R50* genotype exhibited an association with the IL-6 targeting pathway in human gingival cells [49]. An investigation has revealed the functional impact of nonsynonymous variation at site 516 on salicin phenotypic variance in diverse Africans. Additionally, the study found that the majority of other nonsynonymous substitutions have minimal or no effect on cell surface expression in vitro. These findings strongly suggest that a primary polymorphism at TAS2R16 significantly influences salicin recognition [50]. Also, various other scientists, such as Wendell et al., found associations between *TAS2R38* and *TAS1R2* roles in caries risk and/or protection [34]. Also, Gil et al., in their in vitro research, found *TAS2R38* association modulating the reaction of gingival epithelial cells to bacteria associated with both caries and periodontal disease [26]. The researchers Wendell et al. have documented the involvement of the *TAS2R38* haplotype PAV in safeguarding the primary dentition against dental caries [34]. Previous scientific research has demonstrated that the gene *TAS2R38* PAV variant plays a protective role in preventing early colonization of the oral cavity by cariogenic infections [51]. Moreover, it has been observed that the expression of *TAS2R* is subject to fluctuations in response to environmental stimuli such as smoking behavior [52]. Although the expression differs, in a study conducted by Amisten et al., it was demonstrated that the expression of *TAS2R3*, *7*, *14*, *19*, *20*, *31*, *43*, *45*, and *46* was observed in human adipose tissue [53]. Furthermore, the increase in intracellular Ca^2+^ has a role in the downstream actions of TAS2Rs, including the release of antimicrobial peptides (AMPs), clearance of pathogens, and activation of respiratory reflexes [54].The compound epigallocatechin 3-O-gallate, which is the primary flavonoid found in green tea, has been found to activate the *TAS2R144* receptor in white adipose tissue and skeletal muscle [55]. A recent study has shown that the bitter compound denatonium benzoate can reduce periodontitis in mice by activating TAS2Rs receptors. The study also demonstrated that gingival solitary chemosensory cells (gSCCs) play a role in this effect by promoting the release of antimicrobial peptides (AMPs) [33]. It is imperative to conduct future research and undertake a thorough analysis of the potential correlation between polymorphisms in the *TAS2R16* gene and the occurrence of periodontitis. This is necessary in order to facilitate additional investigations and establish comparisons with better-studied gene polymorphisms, such as the *TAS2R38* gene. In comparison, previous studies have demonstrated that variations in the *TAS2R38* gene, specifically single nucleotide polymorphisms (SNPs), have the potential to influence an individual’s susceptibility to respiratory infections, particularly chronic rhinosinusitis (CRS) [56]. As Kim et al. discovered, it is possible that various *TAS2R* alleles exhibit distinct patterns of ligand specificity [57].

When analysing *TAS2R16* family further, researchers Schembre et al. did not find any statistically significant connections between the *TAS2R38* PAV/PAV diplotype or *TAS2R16* (rs846672) polymorphism and the chosen dietary variables [58]. Wolfle et al. conducted research in 2015, and the findings of this study demonstrated that the SH-SY5Y human neuroblastoma cell line exhibits responsiveness to salicin, resulting in the stimulation of neurite outgrowth through a mechanism involving *TAS2R16* and Erk signalling pathways [59]. Nevertheless, other scientists’ work indicates that there is variation in the expression of T2Rs in cells associated with breast cancer [60]. Research by Bona et al. found an association between T/T genotype and longevity in the Calabria population, which was found to be statistically significant and remained significant in the meta-analysis incorporating data from the Cilento population. Hence, the findings of that study provide further support for the proposition that individuals with the *TAS2R16* genotype T/T exhibit a correlation with increased lifespan [61]. Chen et al. have reported their results of expression of *TAS2R,* which have been observed in the arteries of systemic circulation, such as rat mesenteric and cerebral arteries, as well as human omental arteries—the following study presents empirical evidence supporting the existence of *TAS2R* receptors within arterial tissues, suggesting their potential involvement in regulating vascular functions [62]. Another investigation by Kang et al. revealed the presence of *TAS2R* expression in human dental pulp stem cells (hDPSCs). It was observed that *TAS2R* facilitated the differentiation of hDPSCs into odontoblasts by facilitating an elevation in intracellular calcium levels through the conventional signalling pathway of G protein-coupled receptors (GPCRs). This finding suggests that *TAS2R* could serve as a promising target for the development of conservative therapies aimed at effectively repairing dental pulp in an inflammatory microenvironment [63].

The presence of TAS2R16 has been detected in human neural tissue. Salicin, a selective agonist of TAS2R16, has the ability to regulate the development of neurites by activating TAS2R16 [59].

Given the notable abundance of TAS2R16, TAS2R38, TAS2R31, TAS2R39, and TAS2R43 in human gingival fibroblasts (HGF), along with the evident ability of their agonists to stimulate calcium ions, it is reasonable to anticipate that these TAS2Rs have important roles in the periodontium. TAS2R16, among the top five TAS2Rs with the highest expression levels, demonstrated the most potent Ca^2+^-accumulating effects when exposed to salicin. This suggests that TAS2R16 may have regulatory effects on the physiology of human gingival fibroblasts (HGFs) and periodontal health. Due to the widespread presence of TAS2Rs in various cell types, it is believed that gingival fibroblasts also express TAS2Rs. These receptors play a role in reducing excessive inflammatory reactions and help maintain strict control over periodontal inflammation. Zhou’s investigation validated the presence of TAS2Rs and the subsequent activation of signalling components in HGFs. TAS2R16 activation by salicin counteracted the production of cytokines generated by LPS by reducing intracellular cAMP levels and blocking the NF-κB signalling pathway in human gingival fibroblasts (HGFs). Since the activation of TAS2R16 suppresses the inflammatory response in human gingival fibroblasts (HGFs), TAS2Rs could serve as a promising target for the treatment of periodontitis. Additional research is required to examine if the activation of TAS2R16 can mitigate alveolar bone loss in both animal models and clinical populations [14].

These findings represent that the *TAS2R* family and its polymorphisms are associated with various pathologies, including oral diseases, to which our research proves significant correlations between *TAS2R16* rs860170 genotypes TT, CT, and CC polymorphisms and generalized periodontitis. Further studies focusing on *TAS2R16* gene polymorphisms and their potential association with periodontitis are essential to enhance our understanding of the genetic basis of this complex disease. To gain a comprehensive understanding of TAS2R16’s involvement in the etiopathogenesis of generalized periodontitis, additional research involving a larger cohort of patients in the case group and adequate representation from diverse populations would be beneficial. The limited number of patients in our study group was chosen based on a random selection to ensure fairness and unbiased representation for TAS2R16 serum level analysis. However, this limited sample size may contribute to the observed absence of a significant relationship. While we acknowledge the potential relevance of local quantification, such as in gingival crevicular fluid or saliva, our choice was made to enable a standardized and systematic comparison between the two groups. We understand the importance of local quantification and appreciate the reviewer’s perspective. Larger-scale studies are warranted not only to validate our findings but also to explore the potential benefits of local quantification methods in understanding the role of TAS2R16 in periodontal health. Therefore, future investigations with an increased sample size and consideration of diverse populations are necessary to draw conclusive insights and enhance the robustness of our findings.

The study examined TAS2R16 gene variants in individuals with generalised periodontitis, yielding significant insights into the genetic foundation and development of this complex disease. Furthermore, the future scope of TAS2R16 gene studies holds promise for personalized medicine, therapeutic targets, and prognostic markers in the management of generalised periodontitis. Continued research in this field is crucial to furthering our understanding and improving the clinical outcomes for patients affected by this disease.

## 5. Conclusions

Females carrying the *TAS2R16* rs978739 C allele demonstrated a higher susceptibility to the development of generalized periodontitis. Significant correlations were observed between the presence of *TAS2R16* rs860170 polymorphisms, increased *TAS2R16* serum levels, and the onset of generalized periodontitis. The development of generalized PD was found to be associated with the *TAS2R16* rs860170 genotypes TT, CT, and CC in a population aged 70 years or older.

## Figures and Tables

**Table 1:** Demographic characteristics of the study groups.

Characteristics	Group		*p*-Value
	PD (*n* = 280)	Reference Group (*n =* 310)	
Male, *n* (%)	112 (40.0)	143 (46.1)	0.133 ^1^
Female, *n* (%)	168 (60.0)	167 (53.9)	
Age years; median (IQR)	71.0 (18.0)	70.0 (13.0)	0.105 ^2^

PD—periodontitis; IQR—interquartile range; *p*-value—significance level (differences considered significant when *p* < 0.05); ^1^ Pearson chi-square test was used; ^2^ Mann–Whitney U test was used.

**Table 2:** Summarized clinical characteristics of periodontitis patients and healthy subjects.

Periodontitis Patient	Healthy Subject
>18 years old	>18 years old
Generalized periodontitis of stages III and IV—more than 30% of the patient’s oral region affected by periodontitis	No bleeding on probing (BOP)
Radiographic evidence of bone loss	No clinical signs of gingiva inflammation
Interdental clinical attachment loss ≥ 5 mm (III–IVth stage periodontitis)	No clinical attachment loss and probing depth ≤ 3 mm
Tooth loss	No previous history of periodontal diseases
Vertical bone loss ≥ 3 mm	No systemic infectious/non-infectious diseases
<20 remaining teeth (10 opposing pairs)	
Probing depths ≥ 6 mm	

**Table 3:** Distribution of genotypes and alleles of *TAS2R16* rs860170, rs978739, rs1357949 polymorphisms in patients with PD and control group.

Polymorphism	PD, *n* (%)	Reference Group, *n* (%)	HWE *p*-Value	*p*-Value
*TAS2R16* rs860170			<0.0001	
TT	117 (41.8)	120 (38.7)		**<0.001**
CT	163 (58.2)	174 (56.1)		
CC	0 (0)	16 (5.2)		
Total	280 (100)	310 (100)		
Allele				
T	397 (70.9)	414 (66.8)		0.128
C	163 (29.1)	206 (33.2)		
*TAS2R16* rs978739			0.644	
TT	126 (45.0)	155 (50.0)		0.249
CT	117 (41.8)	126 (40.6)		
CC	37 (13.2)	29 (9.4)		
Total	280 (100)	310 (100)		
Allele				
T	369 (65.9)	436 (70.3)		0.103
C	191 (34.1)	184 (29.7)		
*TAS2R16* rs1357949 AA			0.318	
AA	138 (49.3)	135 (43.5)		0.318
AG	114 (40.7)	145 (46.8)		
GG	28 (10.0)	30 (9.7)		
Total	280 (100)	310 (100)		
Allele				
A	390 (69.6)	415 (66.9)		0.319
G	170 (30.4)	205 (33.1)		

PD—periodontitis; *p*-value—significance level (differences considered significant when *p* < 0.05/3).

**Table 4:** Binary logistic regression analysis of *TAS2R16* rs860170, rs978739, rs1357949 in patients with PD and reference groups, when age and gender were included as covariates.

Model	Genotype/Allele	OR (95% CI)	*p*-Value	AIC
*TAS2R16 rs860170*				
Codominant	CT vs. TT	0.984 (0.704–1.375)	0.924	795.155
	CC vs. TT	-	0.998	
Dominant	TC + CC vs. TT	0.910 (0.652–1.269)	0.578	812.518
Recessive	CC vs. TT + CT	-	0.998	793.164
Overdominant	CT vs. CC + TT	1.114 (0.802–1.548)	0.518	812.411
Additive	C	0.776 (0.572–1.055)	0.106	810.197
*TAS2R16 rs978739*				
Codominant	CT vs. TT	1.143 (0.809–1.616)	0.449	812.597
	CC vs. TT	1.495 (0.868–2.575)	0.147	
Dominant	TC + CC vs. TT	1.210 (0.874–1.676)	0.251	811.509
Recessive	CC vs. TT + CT	1.405 (0.836–2.363)	0.200	811.171
Overdominant	CT vs. CC + TT	1.060 (0.762–1.474)	0.731	812.710
Additive	C	1.196 (0.939–1.522)	0.147	810.723
*TAS2R16 rs1357949*				
Codominant	AG vs. AA	0.780 (0.553–1.099)	0.156	812.813
	GG vs. AA	0.889 (0.503–1.573)	0.687	
Dominant	AG + GG vs. AA	0.799 (0.577–1.107)	0.178	811.013
Recessive	GG vs. AA + AG	1.002 (0.580–1.730)	0.994	812.828
Overdominant	AG vs. GG + AA	0.796 (0.573–1.106)	0.174	810.975
Additive	G	0.878 (0.685–1.126)	0.307	811.781

PD—periodontitis; OR: odds ratio; AIC: Akaike information criterion; *p*-value—significance level (differences considered significant when *p* < 0.05).

**Table 5:** The genotypes and alleles of *TAS2R16* rs860170, rs978739, and rs1357949 distribution in PD and reference groups between males.

Polymorphism	PD, *n* (%)	Reference Group, *n* (%)	*p*-Value
*TAS2R16* rs860170			
TT	43 (38.4)	47 (32.9)	**0.002**
CT	69 (61.6)	81 (56.6)	
CC	0 (0)	15 (10.5)	
Total	112 (100)	143 (100)	
Allele			
T	155 (69.2)	175 (61.2)	0.060
C	69 (30.8)	111 (38.8)	
*TAS2R16* rs978739			
TT	57 (50.9)	65 (45.5)	0.482
CT	45 (40.2)	68 (47.6)	
CC	10 (8.9)	10 (7.0)	
Total	112 (100)	143 (100)	
Allele			
T	159 (71.0)	198 (69.2)	0.668
C	65 (29.0)	88 (30.8)	
*TAS2R16* rs1357949			
AA	52 (46.4)	62 (43.4)	0.800
AG	51 (45.5)	71 (49.7)	
GG	9 (8.0)	10 (7.0)	
Total	112 (100)	143 (100)	
Allele			
A	155 (69.2)	195 (68.2)	0.806
G	69 (30.8)	91 (31.8)	

PD—periodontitis; *p*-value—significance level (differences considered significant when *p* < 0.05/3).

**Table 6:** Binary logistic regression analysis of PD and reference groups in males.

Model	Genotype/Allele	OR (95% CI)	*p*-Value	AIC
*TAS2R16 rs860170*				
Codominant	CT vs. TT	0.931 (0.552–1.572)	0.789	335.572
	CC vs. TT	-	0.998	
Dominant	TC + CC vs. TT	0.786 (0.469–1.317)	0.360	350.889
Recessive	CC vs. TT + CT	-	0.998	333.643
Overdominant	CT vs. CC + TT	1.228 (0.742–2.034)	0.424	351.087
Additive	C	0.606 (0.389–0.945)	0.027	344.730
*TAS2R16 rs978739*				
Codominant	CT vs. TT	0.755 (0.450–1.267)	0.267	352.266
	CC vs. TT	1.140 (0.443–2.937)	0.786	
Dominant	TC + CC vs. TT	0.804 (0.490–1.320)	0.388	350.983
Recessive	CC vs. TT + CT	1.304 (0.523–3.251)	0.569	351.404
Overdominant	CT vs. CC + TT	0.741 (0.449–1.222)	0.240	350.340
Additive	C	0.915 (0.617–1.357)	0.660	351.533
*TAS2R16 rs1357949*				
Codominant	AG vs. AA	0.856 (0.512–1.433)	0.555	353.281
	GG vs. AA	1.073 (0.406–2.839)	0.887	
Dominant	AG + GG vs. AA	0.883 (0.537–1.452)	0.624	351.487
Recessive	GG vs. AA + AG	1.162 (0.456–2.965)	0.753	351.629
Overdominant	AG vs. GG + AA	0.848 (0.516–1.392)	0.514	351.301
Additive	G	0.948 (0.636–1.415)	0.795	351.659

PD—periodontitis; OR: odds ratio; AIC: Akaike information criterion; *p*-value—significance level (differences considered significant when *p* < 0.05/3).

**Table 7:** The genotypes and alleles of *TAS2R16* rs860170, rs978739, and rs1357949 distribution in PD and reference groups between females.

Polymorphism	PD, *n* (%)	Reference Group, *n* (%)	*p*-Value
*TAS2R16* rs860170			
TT	74 (44.0)	73 (43.7)	0.604
CT	94 (56.0)	93 (55.7)	
CC	0 (0)	1 (0.6)	
Total	168 (100)	167 (100)	
Allele			
T	242 (72.0)	239 (71.6)	0.893
C	94 (28.0)	95 (28.4)	
*TAS2R16* rs978739			
TT	69 (41.1)	90 (53.9)	0.059
CT	72 (42.9)	58 (34.7)	
CC	27 (16.1)	19 (11.4)	
Total	168 (100)	167 (100)	
Allele			
T	210 (62.5)	238 (71.3)	**0.016**
C	126 (37.5)	96 (28.7)	
*TAS2R16* rs1357949			
AA	86 (51.2)	73 (43.7)	0.374
AG	63 (37.5)	74 (44.3)	
GG	19 (11.3)	20 (12.0)	
Total	168 (100)	167 (100)	
Allele			
A	235 (69.9)	220 (65.9)	0.259
G	101 (30.1)	114 (34.1)	

PD—periodontitis; *p*-value—significance level (differences considered significant when *p* < 0.05/3).

**Table 8:** Binary logistic regression analysis of PD and reference groups in females.

Model	Genotype/Allele	OR (95% CI)	*p*-Value	AIC
*TAS2R16 rs860170*				
Codominant	CT vs. TT	0.997 (0.647–1.536)	0.989	467.010
	CC vs. TT	-	1.000	
Dominant	TC + CC vs. TT	0.986 (0.641–1.519)	0.951	466.402
Recessive	CC vs. TT + CT	-	1.000	465.010
Overdominant	CT vs. CC + TT	1.011 (0.657–1.556)	0.961	466.403
Additive	C	0.964 (0.629–1.477)	0.865	466.377
*TAS2R16 rs978739*				
Codominant	CT vs. TT	1.619 (1.015–2.583)	0.043	462.718
	CC vs. TT	1.854 (0.953–3.606)	0.069	
Dominant	TC + CC vs. TT	1.677 (1.088–2.584)	0.019	460.870
Recessive	CC vs. TT + CT	1.492 (0.794–2.802)	0.214	464.841
Overdominant	CT vs. CC + TT	1.409 (0.906–2.192)	0.128	464.073
Additive	C	1.427 (1.048–1.943)	0.024	461.219
*TAS2R16 rs1357949*				
Codominant	AG vs. AA	0.723 (0.457–1.143)	0.165	466.435
	GG vs. AA	0.806 (0.400–1.626)	0.547	
Dominant	AG + GG vs. AA	0.740 (0.482–1.138)	0.171	464.526
Recessive	GG vs. AA + AG	0.937 (0.481–1.828)	0.849	466.369
Overdominant	AG vs. GG + AA	0.754 (0.487–1.167)	0.205	464.797
Additive	G	0.838 (0.611–1.150)	0.274	465.204

PD—periodontitis; OR: odds ratio; AIC: Akaike information criterion; *p*-value—significance level (differences considered significant when *p* < 0.05/3).

**Table 9:** Genotype and allele distribution of *TAS2R16* polymorphisms (rs860170, rs978739, rs1357949) in patients with PD and reference group: subjects under 70 years of age.

Polymorphism	PD, *n* (%)	Reference Group, *n* (%)	*p*-Value
*TAS2R16 rs860170*			
TT	55 (40.7)	59 (38.8)	0.163
CT	80 (59.3)	89 (58.6)	
CC	0 (0)	4 (2.6)	
Total	135 (100)	152 (100)	
Allele			
T	190 (70.4)	207 (68.1)	0.555
C	80 (29.6)	97 (31.9)	
*TAS2R16 rs978739*			
TT	59 (43.7)	83 (54.6)	0.182
CT	61 (45.2)	55 (36.2)	
CC	15 (11.1)	14 (9.2)	
Total	135 (100)	152 (100)	
Allele			
T	179 (66.3)	221 (72.7)	0.096
C	91 (33.7)	83 (27.3)	
*TAS2R16 rs1357949*			
AA	65 (48.1)	65 (42.8)	0.645
AG	58 (43.0)	71 (46.7)	
GG	12 (8.9)	16 (10.5)	
Total	135 (100)	152 (100)	
Allele			
A	188 (69.6)	201 (66.1)	0.369
G	82 (30.4)	103 (33.9)	

PD—periodontitis; *p*-value—significance level (differences considered significant when *p* < 0.05/3).

**Table 10:** Binary logistic regression analysis of *TAS2R16* rs860170, rs978739, rs1357949 in patients with PD and control groups: subjects under 70 years of age.

Model	Genotype/Allele	OR (95% CI)	*p*-Value	AIC
*TAS2R16 rs860170*				
Codominant	CT vs. TT	0.964 (0.599–1.551)	0.881	395.701
	CC vs. TT	-	0.999	
Dominant	TC + CC vs. TT	0.923 (0.575–1.482)	0.739	398.748
Recessive	CC vs. TT + CT	-	0.999	393.724
Overdominant	CT vs. CC + TT	1.030 (0.643–1.649)	0.903	398.844
Additive	C	0.842 (0.536–1.322)	0.454	398.297
*TAS2R16 rs978739*				
Codominant	CT vs. TT	1.560 (0.952–2.557)	0.078	397.446
	CC vs. TT	1.507 (0.676–3.358)	0.316	
Dominant	TC + CC vs. TT	1.549 (0.972–2.470)	0.066	395.453
Recessive	CC vs. TT + CT	1.232 (0.571–2.657)	0.594	398.575
Overdominant	CT vs. CC + TT	1.454 (0.905–2.335)	0.122	396.453
Additive	C	1.338 (0.942–1.903)	0.104	396.197
*TAS2R16 rs1357949*				
Codominant	AG vs. AA	0.817 (0.501–1.331)	0.417	399.981
	GG vs. AA	0.750 (0.329–1.709)	0.494	
Dominant	AG + GG vs. AA	0.805 (0.505–1.282)	0.361	398.022
Recessive	GG vs. AA + AG	0.829 (0.377–1.822)	0.641	398.640
Overdominant	AG vs. GG + AA	0.859 (0.539–1.370)	0.524	398.453
Additive	G	0.847 (0.592–1.211)	0.362	398.026

PD—periodontitis; OR: odds ratio; AIC: Akaike information criterion; *p*-value—significance level (differences considered significant when *p* < 0.05).

**Table 11:** Genotype and allele distribution of *TAS2R16* polymorphisms (rs860170, rs978739, rs1357949) in patients with PD and reference group: subjects aged 70 and above.

Polymorphism	PD, *n* (%)	Reference Group, *n* (%)	*p*-Value
*TAS2R16* rs860170			
TT	62 (42.8)	61 (38.6)	**0.003**
CT	83 (57.2)	85 (53.8)	
CC	0 (0)	12 (7.6)	
Total	145 (100)	158 (100)	
Allele			
T	207 (71.4)	207 (65.5)	0.121
C	83 (28.6)	109 (34.5)	
*TAS2R16* rs978739			
TT	67 (46.2)	72 (45.6)	0.256
CT	56 (38.6)	71 (44.9)	
CC	22 (15.2)	15 (9.5)	
Total	145 (100)	158 (100)	
Allele			
T	190 (65.5)	215 (68.0)	0.510
C	100 (34.5)	101 (32.0)	
*TAS2R16* rs1357949			
AA	73 (50.3)	70 (44.3)	0.344
AG	56 (38.6)	74 (46.8)	
GG	16 (11.0)	14 (8.9)	
Total	145 (100)	158 (100)	
Allele			
A	202 (69.7)	214 (67.7)	0.608
G	88 (30.3)	102 (32.3)	

PD—periodontitis; *p*-value—significance level (differences considered significant when *p* < 0.05/3).

**Table 12:** Binary logistic regression analysis of *TAS2R16* rs860170, rs978739, rs1357949 in patients with PD and control groups: subjects aged 70 and above.

Model	Genotype/Allele	OR (95% CI)	*p*-Value	AIC
*TAS2R16* rs860170				
Codominant	CT vs. TT	0.961 (0.603–1.530)	0.688	407.380
	CC vs. TT	-	0.999	
Dominant	TC + CC vs. TT	0.842 (0.532–1.332)	0.462	420.949
Recessive	CC vs. TT + CT	-	0.999	405.408
Overdominant	CT vs. CC + TT	1.150 (0.730–1.810)	0.547	421.126
Additive	C	0.683 (0.454–1.029)	0.068	418.124
*TAS2R16* rs978739				
Codominant	CT vs. TT	0.848 (0.523–1.374)	0.502	420.759
	CC vs. TT	1.576 (0.755–3.290)	0.226	
Dominant	TC + CC vs. TT	0.975 (0.620–1.532)	0.911	421.477
Recessive	CC vs. TT + CT	1.705 (0.847–3.431)	0.135	419.210
Overdominant	CT vs. CC + TT	0.771 (0.488–1.219)	0.266	420.249
Additive	C	1.114 (0.801–1.549)	0.522	421.078
*TAS2R16* rs1357949				
Codominant	AG vs. AA	0.726 (0.450–1.170)	0.188	421.350
	GG vs. AA	1.096 (0.498–2.411)	0.820	
Dominant	AG + GG vs. AA	0.785 (0.499–1.233)	0.293	420.382
Recessive	GG vs. AA + AG	1.276 (0.599–2.716)	0.528	421.089
Overdominant	AG vs. GG + AA	0.714 (0.452–1.129)	0.149	419.403
Additive	G	0.914 (0.648–1.289)	0.609	421.227

PD—periodontitis; OR: odds ratio; AIC: Akaike information criterion; *p*-value—significance level (differences considered significant when *p* < 0.05).

**Table 13:** TAS2R16 serum levels associations with *TAS2R16* rs860170, rs978739, rs1357949.

Gene, SNP	Genotype	Serum TAS2R16 Levels		*p*-Value
		PD Group	Reference Group	
*TAS2R16* rs860170	TT	0.112 (0.04)	0.089 (0.03)	0.193 *
	CT	0.147 (0.06)	0.096 (0.03)	0.031 *
	CC	-	-	NA
*TAS2R16* rs978739	TT	0.108 (0.03)	0.093 (0.03)	0.316 *
	CT	0.164 (0.07)	0.092 (0.03)	0.022 *
	CC	0.099 (0.01)	0.088 (0.03)	0.716 *
*TAS2R16* rs1357949	AA	0.144 (0.06)	0.087 (0.02)	0.059 *
	AG	0.125 (0.06)	0.098 (0.04)	0.246 *
	GG	0.097 (0.02)	0.084 (0.03)	0.439 *

* Student *t* test was used, mean (std. deviation) was compared.

**Table 14:** Linkage disequilibrium between the tested *TAS2R16* polymorphisms (rs860170, rs978739, rs1357949) in patients with PD.

SNP-SNP	D′	r^2^	*p*-Value
rs860170—rs978739	0.7703	0.1258	<0.001
rs860170—rs1357949	0.6328	0.0845	<0.001
rs978739—rs1357949	0.9875	0.2116	<0.001

**Table 15:** Haplotype association of *TAS2R16* rs860170, rs978739, rs1357949 with the predisposition to PD occurrence.

Haplotype	*rs860170*	*rs978739*	*rs1357949*	Frequency, %		OR (95% CI)	*p*-Value
				Control Group	PD Group		
1	T	C	A	25.83	33.29	1.00	-
2	T	T	G	28.00	29.17	0.79 (0.58–1.08)	0.140
3	C	T	A	24.85	27.11	0.88 (0.61–1.26)	0.480
4	T	T	A	12.67	8.43	0.53 (0.34–0.81)	**0.003**
5	C	T	A	0.48	1.18	0.18 (0.07–0.49)	**<0.001**
6	C	C	A	3.58	0.82	0.16 (0.05–0.56)	**0.004**

OR—likelihood ratio; PD—periodontitis. *p*-value—significance level (differences considered significant when *p* < 0.05).

## Data Availability

The original contributions presented in the study are included in the article, further inquiries can be directed to the corresponding author/s.
